# Can Clinical Pathways enhance the implementation of a Casemix system? A case study in a teaching hospital in Malaysia

**DOI:** 10.1186/1472-6963-11-S1-A6

**Published:** 2011-10-19

**Authors:** SM Aljunid, A Ismail, S Sulong

**Affiliations:** 1 UNU-IIGH, UNU-International Institute For Global Health, Kuala Lumpur, Federal Territory, 5600, Malaysia; 2 Dept of Community Health, University Kebangsaan Malasyaia, Kuala Lumpur, Federal Territory, 5600, Malaysia; 3 International Casemix and Clinical Coding Centre, University Kebangsaan Malasyaia, Kuala Lumpur, Federal Territory, 5600, Malaysia

## Introduction

A Clinical Pathway (CP) is a multidisciplinary plan of care based on best clinical practice for a specified group of patients with a particular diagnosis. A CP is designed to minimize delays, optimize resource utilization, and maximize quality of care. CPs support the implementation of Casemix by reducing variations of care, increasing homogeneity of cases, improving quality of Casemix data, and enhancing costing analysis in Casemix.

## Methods

Universiti Kebangsaan Malaysia Medical Centre (UKMMC) in collaboration with United Nations University International Institute For Global Health (UNU-IIGH) has developed, implemented, and evaluated four clinical pathways. These are ST Elevation Myocardial Infarction (STEMI); Chronic Obstructive Pulmonary Diseases (COPD); Elective Lower Segment Caesarean Section (LSCS); and Elective Total Knee Replacement (TKR).

This non-randomized, single-blind, controlled study used enrolled patients from January 2008 to December 2008 as a control group (non-CP group). CP was assigned to all new patients admitted with the above diseases from the year 2009 until 2010.

## Results

There was a significant reduction in the average length of stay (ALOS) of the COPD CP group (5.85 ±1.92 days) when compared to the non-CP group (7.31 ±2.75 days, Z= -3.893, P<0.001). In STEMI, the ALOS for patients in the non-CP group was 8.15 ±2.25 days, while in the CP group it was 5.52 ±1.42 days (t = -4.85, P<0.001). There was also a significant difference in ALOS in LSCS, with the CP group staying 4.04 ±0.61 days compared to the non-CP group staying 4.99 ±2.94 (Z = -3.221, P<0.001). In TKR, though, there was no significant difference between the ALOS of the CP and non-CP groups (9.93 ±4.32 days vs. 9.05 ±3.59 days). However, the age of the patient, co-morbidity, readmission, and complication rates did not differ significantly between CP and non-CP groups.

## Conclusions

There was significantly shorter ALOS among patients in CP groups compared to non CP groups – except for TKR. In general, the implementation of CPs has had a positive impact in increasing the homogeneity of cases being managed in UKMMC. Hence, we conclude that the use of Clinical Pathways has enhanced and supported the implementation of the Casemix system in the hospital.

